# Loss of the α2β1 Integrin Alters Human Papilloma Virus-Induced Squamous Carcinoma Progression *In Vivo* and *In Vitro*

**DOI:** 10.1371/journal.pone.0026858

**Published:** 2011-10-27

**Authors:** Thuy Tran, Brittney Barlow, Lynda O'Rear, Brenda Jarvis, Zhengzhi Li, Kent Dickeson, William Dupont, Mary Zutter

**Affiliations:** 1 Department of Pathology, Vanderbilt University School of Medicine, Nashville, Tennessee, United States of America; 2 Department of Cancer Biology, Vanderbilt University School of Medicine, Nashville, Tennessee, United States of America; 3 Department of Biostatistics, Vanderbilt University School of Medicine, Nashville, Tennessee, United States of America; Wistar Institute Program, United States of America

## Abstract

Expression of the α2β1 integrin, a receptor for collagens and laminin, is altered during tumor progression. Recent studies have linked polymorphisms in the α2 integrin gene with oral, squamous cell carcinoma (SCC). To determine the α2β1 integrin's role in SCC progression, we crossed α2-null mice with K14-HPV16 transgenic animals. Pathological progression to invasive carcinoma was evaluated in HPV-positive, α2-null (HPV/KO) and HPV-positive, wild-type (HPV/WT) animals. α2β1 integrin expression stimulated progression from hyperplasia and papillomatosis to dysplasia with concomitant dermal mast cell infiltration. Moreover, lymph node metastasis was decreased by 31.3% in HPV/KO, compared to HPV/WT, animals. To evaluate the integrin-specific impact on the malignant epithelium versus the microenvironment, we developed primary tumor cell lines. Although transition from dysplasia to carcinoma was unaltered during spontaneous tumor development, isolated primary HPV/KO SCC cell lines demonstrated decreased migration and invasion, compared to HPV/WT cells. When HPV/WT and HPV/KO SCC cells were orthotopically injected into WT or KO hosts, tumor α2β1 integrin expression resulted in decreased tumor latency, regardless of host integrin status. HPV/WT SCC lines failed to demonstrate a proliferative advantage *in vitro*, however, the HPV/WT tumors demonstrated increased growth compared to HPV/KO SCC lines *in vivo.* Although contributions of the integrin to the microenvironment cannot be excluded, our studies indicate that α2β1 integrin expression by HPV-transformed keratinocytes modulates SCC growth and progression.

## Introduction

Cancers arise from the accumulation of genetic mutations that alter cell proliferation, differentiation, and tissue organization. Infection with Human Papilloma Virus (HPV) causes 100% of cervical cancer, 90% of anal cancer, 40% of vulvar and vaginal cancer, 15%–35% of oropharyngeal cancers, and approximately 3% of oral cancers [1], [2], [3]. Approximately 6.2 million new HPV infections occur each year globally, with 20 million women currently infected. Cervical cancer is the 7^th^ most common cause of death in women worldwide. Arbeit, Coussens, and Hanahan developed a transgenic mouse model of epithelial carcinogenesis in which the HPV 16 early region genes were expressed in basal keratinocytes under the control of the keratin 14 promoter [4], [5], [6], [7], [8], [9], [10], [11], [12], [13], [14], [15], [16]. This model of squamous epithelial carcinogenesis mimics viral-induced tumor progression in humans.

Cancer progression and metastasis do not solely rely upon the genetic and epigenetic events within the tumor cell, but also on changes in the microenvironment [17], [18], [19]. Integrins mediate both cell-extracellular matrix (ECM) and cell-cell adhesion [20], [21], [22], [23], [24], [25], [26], [27]. As mediators of cell adhesive behavior, integrins play a critical role in tumor progression and metastasis [21], [28]. The α2β1 integrin, primarily a collagen and laminin receptor, is highly expressed on basal keratinocytes where it is involved in adhesion to basement membrane collagens and migration on laminin 5. The integrin is also expressed on many epithelial cells, activated endothelial cells, and some inflammatory cells [4], [5], [6], [29], [30]. Previous studies suggest that α2β1integrin expression is altered *in vivo* during progression of breast, lung, and prostate cancers [31], [32], [33], [34], [35]. Recent studies have linked polymorphisms in the α2β1 integrin with oral, squamous cell carcinoma (SCC) [36]. Dyce *et al*. demonstrated that human SCC cell lines that expressed high levels of the α2β1 integrin were more invasive than cells with low integrin expression [37].

To determine the role of α2β1 integrin expression in squamous epithelial carcinogenesis, we chose the K14-HPV16 model for several reasons: 1. Expression of the α2β1 integrin on squamous epithelial cells is regulated in a differentiation-dependent manner. 2. The K14-HPV16 model investigates the complex interplay between the malignant cells and the host immune system, including a requirement for mast cells in the progression towards invasive SCC. 3. Prior data from our laboratory demonstrated α2β1 integrin expression on connective tissue mast cells and showed that the integrin participated in mast cell activation in response to specific pathogens. 4. HPV-stimulated carcinoma is highly relevant to human disease and represents a significant public health burden.

We now show that α2β1 integrin expression promotes early preneoplastic dysplasia from hyperplasia and papillomatosis to dysplasia. Decreased dysplasia in the HPV/KO mice was associated with decreased recruitment of mast cells at early time points. Although, loss of α2β1 integrin expression did not affect tumor latency, prevalence, tumor growth, or histologic grade, metastasis to the regional lymph nodes was decreased by 31.3%. Since these studies were conducted in animals in which the α2β1 integrin was globally deleted, primary tumor cell lines were developed. Isolated, primary HPV/WT, but not HPV/KO SCC cell lines, migrated rapidly and invaded through a matrix of type I collagen. Following orthotopic injection of HPV/WT and HPV/KO cells into either WT or KO mice, HPV/WT SCC cells formed tumors with a short latency and rapid growth. In contrast, HPV/KO SCC cells either failed to form tumors or grew significantly slower than the HPV/WT tumor cells. Integrin status of the host animal did not influence tumor development. Therefore, our data from both the spontaneous *in vivo* and orthotopic primary tumor cell transplantation *in vivo* models support a role for α2β1 integrin expression by the HPV oncogene-transformed SCCs in malignant progression. The differences between the spontaneous model and the orthotopic injection model suggest that many factors play a role in tumor progression *in vivo*.

## Materials and Methods

### Ethics Statement

Mice were housed in pathogen-free conditions at Vanderbilt University Medical Center in strict compliance with national and institutional animal welfare regulations; all animal experiments were approved by Vanderbilt's IACUC protocol M/10/002.

### Animals and Tumor Measurement

The α2β1 integrin-deficient mice on the FVB/N background were crossed with congenic K14-HPV16 transgenic mice, a generous gift from Lisa Coussens, to generate K14-HPV16, α2-null (HPV/KO) or wild-type (HPV/WT) mice [38]. Non-K14-HPV16-transgenic littermates were used as controls. Mice were monitored weekly for tumor development; tumor volume was calculated as V = 0.52×A×B^2^, where V is the volume, A is the largest diameter, and B is the shortest diameter. Once tumors reached a maximum diameter of at least 10 mm, affected animals were sacrificed, and tumors, blood, ears, and superficial lymph nodes were harvested.

### Histology

Tumor and ear morphology was evaluated on formalin-fixed, paraffin-embedded, and hematoxylin and eosin stained sections. Tumors were classified according to the WHO system of differentiation (1+  =  well, 2+  =  moderate, 3+  =  poorly, and 4+  =  anaplastic/spindle cell) [8]. Ear skin was analyzed for the presence or absence of hyperplasia, papillomatosis, or dysplasia. The number of toluidine blue-stained mast cells was quantitated using the Metamorph imaging system (Molecular Devices, Sunnyvale, CA) [39].

### Immunohistochemistry and Immunofluorescence

Immunohistochemical identification of wide-spectrum cytokeratin expressing metastatic tumor cells was performed on lymph node sections using anti-bovine WSCK antibody, EnVision+ polyclonal labeled polymer, and DAB substrate (all from Dako, Carpinteria, CA). Immunofluorescence analysis of primary tumor cells was conducted on cells plated on coverslips with anti-LYVE-1 (Abcam, Cambridge, MA), anti-WSCK (Dako), and DAPI (Invitrogen). Secondary antibodies included Alexa Fluors 568 and 488 (Invitrogen). Imaging was performed using a Nikon Eclipse 80i microscope and quantitated using ImageJ (NIH, Bethesda, MD).

### Flow Cytometry

Flow cytometric analyses of peripheral blood and infiltrates into ear skin and tumors were performed on a 3-laser, BD LSR II Flow Cytometer in the Vanderbilt Medical Center Flow Cytometry Shared Resource, and data analysis was performed using FlowJo (Tree Star, Inc., Ashland, OR). Tumor-bearing animals were sacrificed and tumor, ear, and peripheral blood leukocytes were collected and processed, as described previously [15]. Cell suspensions were evaluated with either pre-conjugated BD Pharmingen antibodies against mouse Gr-1 or NK1.1, eBioscience antibodies (San Diego, CA) against mouse c-kit, CD11b, F4/80, CD3e, B220, CD4, CD25, or Foxp3, or appropriate isotype controls. Expression of the α2 integrin subunit on primary SCC cells was determined by flow cytometric analysis using anti-CD49b (BD Pharmingen) or IgG control (BD Pharmingen).

### Cell Culture

#### Isolation of Primary Tumor Cells

Primary tumors were dissected under sterile conditions, finely minced, and cultured in primary tumor media containing DMEM-F12 (Mediatech, Inc., Manassas, VA) supplemented with 5% FBS (Atlanta Biologicals, Lawrenceville, GA), 0.01 µg/mL human EGF (Pepro Tech, Rocky Hill, NJ), 1 µg/mL hydrocortisone (Sigma-Aldrich, St. Louis, MO), 0.1 µg/mL cholera toxin (Calbiochem, La Jolla, CA), 0.3 units of insulin (Novo Nordisk, Princeton, NJ), and 100 units of penicillin/streptomycin (Mediatech, Inc.) at 37°C in 5% CO_2_.

#### Proliferation Assay

HPV/WT or HPV/KO tumor cells (2×10^3^) were plated on either collagen type I (BD Biosciences Discovery Labware, Bedford, MA) (100 µg/mL), fibronectin (BD Biosciences Discovery Labware) (100 µg/mL), or plastic and incubated in primary tumor media. Cell proliferation was evaluated every 24 hours using the CellTiter 96 AQueous Non-Radioactive Cell Proliferation Assay (Promega Corporation, Madison, WI), according to the manufacturer's protocol. Plates were read at 490 nm on a Molecular Devices Emax Precision Microplate Reader, and data was analyzed using Softmax (Molecular Devices).

#### Transfection with the α2 Integrin Subunit

The mouse full-length α2 (mα2) integrin subunit (NCBI GenBank Z29987), a gift from Jeffrey Bergelson, was inserted into the mammalian pSRα expression vector. Clones were selected and verified by sequencing. HPV/KO-2 cells were transfected with 20 µg of either pSRα-mα2 or empty pSRα vector using Lipofectamine 2000 (Invitrogen, Carlsbad, CA) according to manufacturer's protocol.

#### Adhesion Assay

The ability of primary tumor cells (1.7×10^4^ cells/well) to adhere at 37°C for 1 hour to either collagen type I (20 µg/mL) or fibronectin (20 µg/mL) was determined using established protocols in the presence of 2 mM MgCl_2_ (Sigma-Aldrich) or 2 mM EDTA (Sigma-Aldrich) [40]. The optical density of the plate was read at 405 nm on a Molecular Devices Emax Precision Microplate Reader, and data was analyzed using Softmax (Molecular Devices).

#### Migration and Invasion Assay

Cell migration and invasion assays were performed using a modification of the protocol described previously [41]. HPV/WT or HPV/KO primary tumor cells (2×10^4^) in primary tumor media containing 1.5% FBS were placed on the upper membrane of 0.8 µm pore transwell filters (Corning Incorporated Life Sciences, Lowell, MA), which was either uncoated or coated with collagen type I (100 µg/mL). Primary tumor media containing 5% FBS was placed in the lower chamber. The number of cells migrating to the lower chamber at 37°C after 18 hours was determined by counting the number of cells in 3 random high power (10X magnification) fields.

### Orthotopic Injection of Primary Tumor Cells

Tumor cells (1×10^6^) derived from two separate HPV/WT or HPV/KO lines were injected subcutaneously into the interscapular region of either WT or KO, 4-5-week-old FVB/N hosts. Tumors were measured twice a week; volumes were calculated as previously described.

### Statistical Analyses

Statistics were performed using student's t-tests, unless otherwise noted. Contingency table analyses with tests for trend were used to analyze all distributions of tumor grades and tumor multiplicity. Tumor volume growth curves were analyzed by spaghetti plots with curve regression analysis to determine average slopes. Chi-squared tests were performed to determine significance of preneoplastic ear histology. Mann-Whitney U tests were performed for analyses of mast cell ear histology, and for analyzing specific groups within inflammatory populations in all flow cytometry data. P-values of ≤0.05 were considered significant. Analyses were performed using Stata (StataCorp LP, College Station, TX) and GraphPad Prism (La Jolla, CA).

## Results

### The α2β1 Integrin Promotes HPV-induced Squamous Epithelial Dysplasia

**Figure 1:**
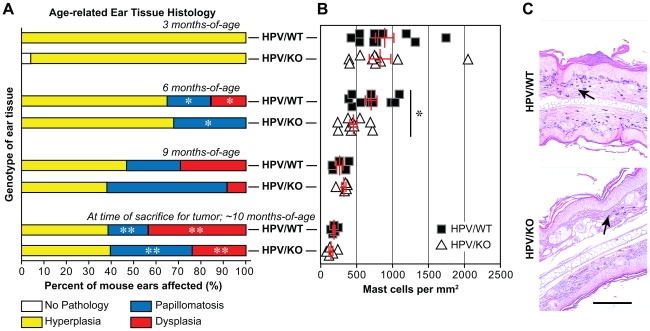
Loss of the α2β1 integrin enhanced HPV-induced papillomatosis but limited dysplasia and preneoplastic mast cell infiltration. *A*, The percentage of HPV/WT and HPV/KO animals with hyperplasia, papillomatosis, or dysplasia at 3-, 6-, and 9-months-of-age, and at sacrifice was determined by morphological examination of ear tissue. The incidence of papillomatosis was significantly increased, while dysplasia was significantly decreased in HPV/KO animals, compared to age-matched, HPV/WT littermates, at 6-months-of-age and at sacrifice (3-months p = 0.304, HPV/WT n = 28, HPV/KO n = 27; 6-months p = 0.0384, HPV/WT n = 20, HPV/KO n = 25; 9-months p = 0.0637, HPV/WT n = 17, HPV/KO n = 13; time-of-sacrifice p = 0.00169, HPV/WT n = 95, HPV/KO n = 68). *B*, Mast cell infiltration into the ear dermis of HPV/WT and HPV/KO animals was quantitated at 3-, 6-, and 9-months-of-age, and at sacrifice. Ear skin of HPV/WT and HPV/KO animals at 3-months-of-age have similar numbers of mast cells (p = 0.58, n = 10 for both groups). At 6-months, HPV/KO ears had decreased numbers of mast cells compared to age-matched HPV/WT littermates (p = 0.019, n = 10 for both groups). Over time, dermal mast cell infiltration decreased. The number of mast cells in the ear skin of HPV/WT and HPV/KO animals was similar at 9 months and at sacrifice (9 months p = 0.32 , n = 5 for both groups; time of sacrifice p = 0.23, n = 5 for both groups). Bars represent mean ± SEM of 3 random images per tissue sample. *C*, A representative toluidine blue-stained section of HPV/WT and HPV/KO premalignant ear tissue at 6 months. Arrows indicate toluidine blue positive cells. Scale bar  = 200 µm.

HPV-induced squamous carcinogenesis involves the step-wise progression from hyperplasia to papillomatosis, to dysplasia, to carcinoma in situ (CIS) and finally to invasive and metastatic cancer [10], [12], [14], [42], [43], [44], [45]. To define the role of the α2β1 integrin in a multi-step, inflammation-driven epithelial carcinogenesis model, we crossed the α2β1 integrin-deficient mouse on an *FVB/n* background with congenic K14-HPV16 mice to generate K14-HPV16/wild-type (HPV/WT) and K14-HPV16/α2-null (HPV/KO) mice. Preneoplastic progression, including hyperplasia, papillomatosis, or dysplasia, was defined in the ear skin of HPV/WT and HPV/KO mice at 3-, 6-, or 9-months-of-age, or at the time of sacrifice due to the development of invasive, squamous carcinoma at another location. By 6-months-of-age, there were significant differences in dysplasia and papillomatosis between the two genotypes: approximately 15% of HPV/WT animals (n = 20), but none of the HPV/KO animals (n = 25), developed dysplasia. In contrast, the incidence of papillomatosis was almost double in the HPV/KO animals (p = 0.0384). Differences in papillomatosis and dysplasia between HPV/KO and HPV/WT ears were also present at later time points (9-months p = 0.0637; time of sacrifice p = 0.00169) (Figure 1A).

Inflammation has been shown to be responsible for driving neoplastic progression in K14-HPV16 transgenic animals [16]. Therefore, the recruitment of inflammatory cells to the skin of HPV/WT and HPV/KO animals at early time points was investigated. There was no significant difference in the total number of CD45-positive cells recruited to the dermis of HPV/WT and HPV/KO mice at either 3- or 6-months-of-age (p = 0.29 and 0.90, respectively; data not shown). At 3-months-of-age, there was also no difference in the number of dermal mast cells in HPV/WT and HPV/KO mouse ears (p = 0.58). In contrast, by 6-months-of-age, there were significantly fewer resident mast cells in HPV/KO than in HPV/WT ears (p = 0.019). Mast cell numbers decreased in ear tissue over time but were similar at 9-months-of-age and at the time of sacrifice between HPV/WT and HPV/KO ears (n = 0.32 and 0.23, respectively) (Figure 1B and 1C). While the quantity of acute mast cells was altered in the preneoplastic ears of K14-HPV16 transgenic mice, detailed studies examining inflammatory populations at the time of animal sacrifice revealed that chronic inflammation is not substantially altered in blood, non-tumorigenic ear, or tumor tissue with integrin loss. In this inflammation-driven tumor model, immune cell differences were dependent on presence of the K14-HPV16 transgene, but ultimately, the α2β1 integrin contributes minimally to long-term, chronic inflammation (Figure S1 and Table S1).

### The α2β1 Integrin Regulates Development of Sebaceous Adenocarcinoma But Not Invasive Squamous Cell Carcinoma

**Figure 2:**
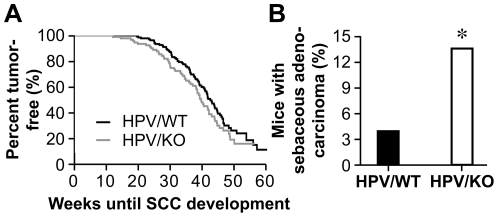
Expression of the α2β1 integrin modulates the incidence of sebaceous adenocarcinoma formation, but not SCC. *A*, Kaplan-Meier plots of tumor-free HPV/WT and HPV/KO mice. Tumor development was recorded when a visible tumor nodule formed. Latency (time to tumor development) was similar in HPV/WT (n = 146) and HPV/KO (n = 94) mice (p = 0.11). *B,* The percentage of HPV/WT and HPV/KO animals that developed either SCC or sebaceous adenocarcinoma was determined morphologically. Development of sebaceous adenocarcinoma was significantly increased in HPV/KO animals (n = 80) compared to HPV/WT mice (n = 100) (p = 0.028).

To determine the impact of α2β1 integrin expression on progression from dysplasia to invasive carcinoma, tumor latency and prevalence in HPV/KO and HPV/WT animals were determined. Tumor latency was similar in HPV/KO and HPV/WT animals (p = 0.11) (Figure 2A). No differences exist in SCC development between HPV/WT (49.4%) and HPV/KO (58.9%) mice by 10-months-of-age (n = 170 and 107, respectively; p = 0.12). The tumor growth rate, number of tumors per animal, and anatomic location of the SCCs were indistinguishable in HPV/KO animals, as compared to HPV/WT mice (Figure S2A, S2B, and data not shown). Therefore, although α2β1 integrin expression promotes epithelial dysplasia, expression does not stimulate tumor progression from dysplasia to invasive carcinoma in the HPV-stimulated model of squamous cancer.

Previous studies demonstrated that α2β1 integrin expression may be associated with normal, regulated, epithelial differentiation and that altered expression of the integrin may be seen in different subtypes of cancer. To determine whether α2β1 integrin expression or lack thereof affected tumor cell differentiation, invasive HPV/WT and HPV/KO tumors were analyzed histologically. SCC differentiation was graded based on the Broder's four-tier system [8]. There was no difference in tumor grade between HPV/WT and HPV/KO animals (p = 0.57), suggesting that the α2β1 integrin did not ultimately impact squamous differentiation in the K14-HPV16 background (Figure S2C). Even though the majority of tumors arising in the wild-type K14-HPV16 background were SCCs, occasionally, these animals developed sebaceous adenocarcinomas, either alone or in areas with concomitant SCC growth [46]. In HPV/KO mice, compared to HPV/WT mice, pure sebaceous adenocarcinomas represented 13.75% versus 4.00% of the tumors, respectively (p = 0.028) (Figure 2B).

### Loss of the α2β1 Integrin by HPV-Induced SCC Decreases Lymph Node Metastasis

**Figure 3:**
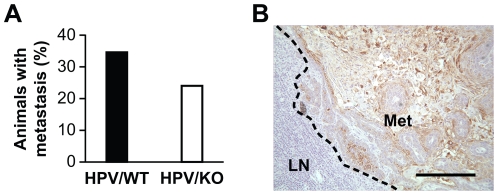
α2β1 integrin expression promoted lymph node metastasis. *A*, The percentage of HPV/WT and HPV/KO animals with SCC that developed regional lymph node metastasis was determined by morphologic and immunohistochemical analysis of superficial lymph nodes. The HPV/KO animals with tumors (n = 71) developed metastasis in 23.9% of cases; HPV/WT animals with tumors (n = 92) developed metastasis in 34.8% of cases (p = 0.14; odds ratio 1.7). *B*, Representative section of a regional lymph node from an HPV/WT animal evaluated by immunohistochemistry for detection of WSCK. The metastatic tumor cells express WSCK (Met) and are surrounded by normal lymph node parenchyma (LN). Scale bar = 200 µm.

Previous studies have shown that approximately 30% of SCCs in the K14-HPV16 mouse metastasize to regional lymph nodes [46]. Consistent with the literature, in our study, 34.8% of HPV/WT tumors metastasized. In contrast, only 23.9% of HPV/KO SCCs metastasized to the lymph nodes (Figure 3A). The presence of lymph node metastasis was verified by immunohistochemical staining for cytokeratin (Figure 3B). Therefore, although there was no difference in tumor growth or tumor latency, expression of the α2β1 integrin promoted tumor metastasis to regional lymph nodes. The difference in metastasis between HPV/WT and HPV/KO animals was not statistically significant (p = 0.14) due to limitations of study size. However, the incidence of lymph node metastasis in HPV/KO mice was decreased by 31.3%, compared to metastasis in the HPV/WT animals. The odds ratio for developing lymph node metastasis in the HPV/WT animals relative to HPV/KO mice was 1.7 (HVP/KO mice 95% confidence interval is 18.3–54.3%; HPV/WT 95% confidence interval is 34.7–81.9%).

### α2β1 Integrin Expression by Squamous Carcinoma Drives Migration and Invasion

**Figure 4:**
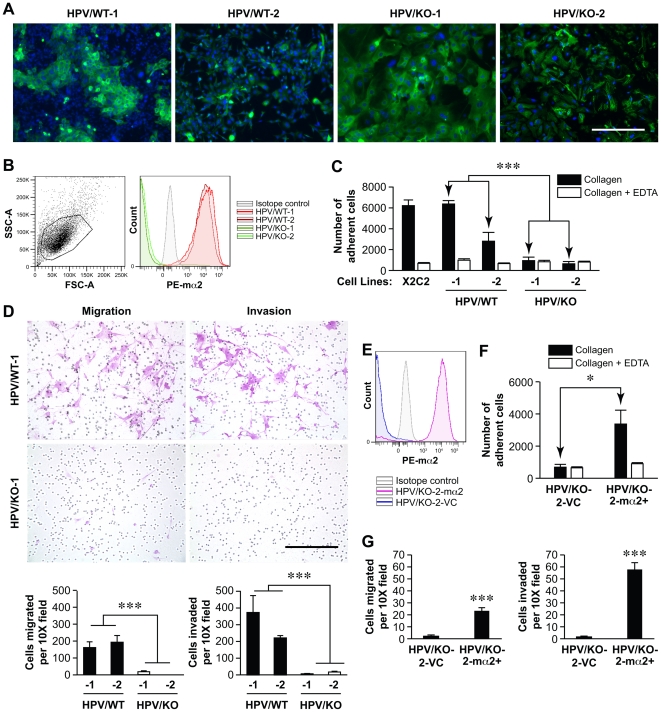
Expression of the α2β1 integrin stimulates SCC migration and invasion in vitro. *A*, Primary HPV/WT and HPV/KO tumor cell lines were stained with anti-WSCK to demonstrate the epithelial origin of the cells. *B*, Flow cytometric analysis using an α2 subunit antibody verified integrin expression on wild-type SCC lines, HPV/WT-1 and -2, and absence of integrin expression on α2-null lines HPV/KO-1 and -2. *C*, HPV/WT-1 and -2 SCC lines adhered to type I collagen in a Mg^2+^-dependent and EDTA-inhibited manner. The X2C2 control cells that express human full-length α2 cDNA served as a positive control. The HPV/KO-1 and -2 cells failed to adhere to type I collagen (p<0.0001). Bars represent mean ± SEM of 2 experiments, performed in duplicate. *D*, HPV/WT-1 and -2 cells exhibited significantly enhanced migration and invasion compared to HPV/KO-1 and -2 cells, cells (p<0.0001 and p<0.0001, respectively). Bars represent mean ± SEM of 3 random photos of transwell experiments, performed in duplicate. *E*, Transfection of the HPV/KO-2 line with pSRα vector containing the wild-type mouse α**α**2 integrin subunit (HPV/KO-2-mα**α**2) restored integrin levels to that found in wild-type SCC cells, as determined by flow cytometric analysis. *F*, Expression of the transfected mα**α**2 subunit in HPV/KO-2-mα**α**2 cells rescued their ability to adhere to collagen, compared to empty vector control transfectants (HPV/KO-2-VC) (p = 0.015). Bars represent mean ± SEM of 2 experiments, performed in duplicate. *G,* The ability of the HPV/KO-2-mα2 transfectants to migrate and invade was restored, compared to HPV/KO-2-VC cells (p = 0.0002 and p<0.0001, respectively). Bars represent mean ± SEM of 3 random photos of transwell experiments, performed in duplicate.

To begin dissecting integrin-dependent changes in the tumor cells versus by cells of the host microenvironment, we focused on the contribution of α2β1 integrin expression by the malignant epithelial cells in tumor progression. Primary tumor cells from HPV/WT and HPV/KO tumors were harvested and two HPV/WT (HPV/WT-1 and HPV/WT-2) and two HPV/KO (HPV/KO-1 and HPV/KO-2) squamous carcinoma cell lines were developed. The epithelial origin of the tumor cells was confirmed by cytokeratin staining (Figure 4A). The HPV/WT, but not the HPV/KO primary tumor cell lines expressed the α2β1 integrin, as determined by flow cytometric analysis (Figure 4B). Both HPV/WT cells, but not the HPV/KO cells, adhered to type I collagen in a Mg^2+^ dependent and EDTA^2+^-inhibitable manner, as did a positive control, NMuMG-X2C2 (derived from the NMuMG3 line stably transfected with full length human α2 integrin subunit) (Figure 4C) [40]. All cells adhered to fibronectin (data not shown). Both HPV/WT and HPV/KO cells proliferated at a similar rate on collagen, fibronectin, or plastic (p = 0.35, p = 0.33, and p = 0.42, respectively) (Figure S3). Therefore, integrin expression did not alter tumor cell proliferation of HPV-driven squamous tumor cells. Although presence of the α2β1 integrin did not alter cell proliferation, expression of the integrin stimulated cell migration and cell invasion *in vitro.* HPV/WT, but not HPV/KO, cells robustly migrated *in vitro* in a three-dimensional transwell migration assay (p<0.0001) and invaded through a barrier of type I collagen (p<0.0001) (Figure 4D).

To determine if α2β1 integrin expression alone could mediate the migratory ability of HPV/KO cell lines, expression of the α2β1 integrin in the HPV/KO-2 cell line was rescued by transfection with a murine α2-integrin subunit expression vector (HPV/KO-2-mα2^+^) or control vector (HPV/KO-2-VC). As determined by flow cytometric analysis, HPV/KO-2-mα2^+^ cells expressed high levels of the murine α2β1 integrin (Figure 4E). Re-expression of the α2 integrin subunit restored the ability of the HPV/KO-2-mα2^+^ cells to adhere to type I collagen in a Mg^2+^ dependent and EDTA^2+^-inhibitable manner, when compared to HPV/KO-2-VC cells (p = 0.015) (Figure 4F). Restoration of murine α2-integrin expression by HPV/KO-2 SCCs also rescued the migratory and invasive ability of the tumor cells through type I collagen, when compared to the control transfectants (p = 0.0002 and p<0.0001, respectively) (Figure 4G).

### α2β1 Integrin Expression by Squamous Epithelium Promotes Tumor Growth In Vivo

**Figure 5:**
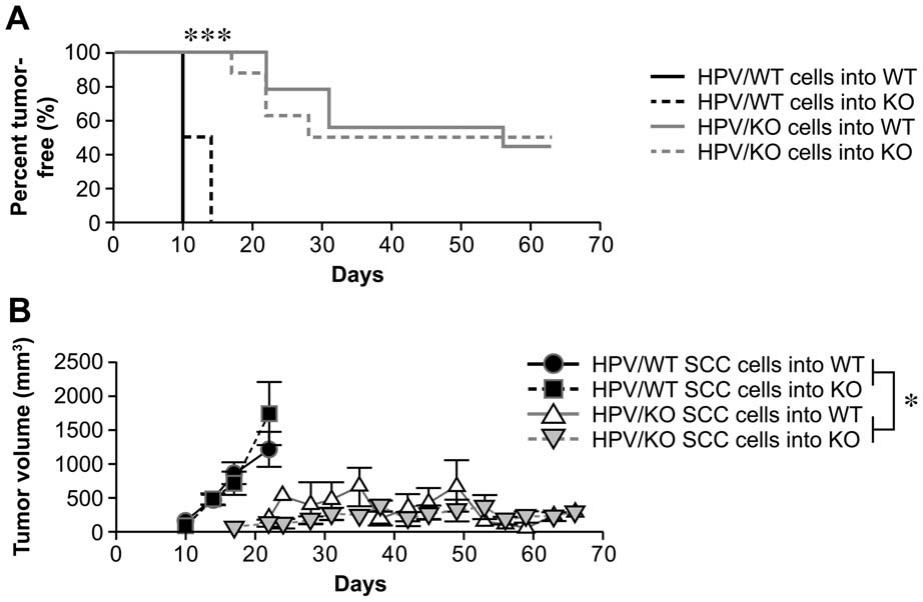
Tumor-specific expression of the α2β1 integrin caused rapid tumor formation and increased tumor growth in vivo, independent of host microenvironment. *A*, Orthotopic injections of SCC lines HPV/KO-1 and -2 into either non-K14-HPV16 transgenic, WT or α2-null (KO) hosts resulted in increased tumor latency by approximately 5–10 days, as compared to SCC lines HPV/WT-1 and -2 (p = 0.0003). Host integrin status had no impact on tumor formation. Tumor latency was dependent on presence of the α2β1 integrin by the tumor cells (n = 4 WT or 4 KO hosts each for HPV/WT-1, HPV/WT-2, and HPV/KO-1 injections; n = 4 WT and 5 KO hosts for HPV/KO-2 SCC cell injections). *B*, Orthotopically injected HPV/KO tumor cells demonstrated decreased growth rate regardless of host integrin status, compared to HPV/WT SCC lines (p = 0.034). Bars represent mean ± SEM.

To determine the impact of α2β1 integrin expression by the tumor cells on tumor growth and latency, the primary tumor cell lines derived from HPV/WT and HPV/KO animals (HPV/WT-1 and -2 and HPV/KO-1 and -2) were orthotopically injected into nontransgenic, wild-type or α2-null mice. The HPV/WT tumor cells grew rapidly when placed in either wild-type or α2-null mice. In contrast, the HPV/KO tumor cells demonstrated increased latency (p = 0.0003) and markedly decreased tumor growth rates (p = 0.034) when compared to mice injected with HPV/WT SCC cells, regardless of recipient mouse integrin status (Figure 5A and 5B). The short time span of orthotopic tumor growth was not permissive for the development of spontaneous metastasis. These results demonstrate that the α2β1 integrin expression promotes tumor growth and progression of SCC in a manner independent of the host microenvironment.

## Discussion

Using the K14-HPV16 cancer model, we demonstrate that lack of α2β1 integrin expression results in decreased progression from epithelial papillomatosis to dysplasia, increased formation of sebaceous adenocarcinomas instead of SCCs, and modestly decreased lymph node metastasis. Although global loss of the α2β1 integrin in all HPV/KO mouse cells did not affect tumor latency, growth, or multiplicity *in vivo,* primary tumor cells derived from HPV/KO animals demonstrated diminished cell migration and invasion *in vitro* and decreased tumor formation and growth when implanted orthotopically into non-K14-HPV16 transgenic wild-type or α2-null animals. Additionally, the host's integrin status did not impact tumor formation or growth, thereby suggesting that α2β1 integrin expression by the tumor microenvironment is not responsible for tumor progression in this model.

Diminished epithelial dysplasia and enhanced papillomatosis in HPV/KO mice suggest that the α2β1 integrin plays a role in regulating epithelial differentiation and promoting the initial steps of neoplasia. The mast cell reduction in 6-month-old HPV/KO mice may promote papillomatosis. On one hand, the reduction in mast cells may limit the further progression of papillomas to carcinoma. On the other hand,mast cell deficient animals have been shown to be more susceptible to papilloma formation than their wild-type counterparts in other models [47]. Therefore, while these inflammatory cells help drive the hyperplasia and dysplasia associated with squamous carcinogenesis, they may be affecting rates of papillomatosis differently [10]. At the stage of invasive carcinoma, neither tumor latency, growth, or differentiation, i.e. grade, was different in HPV/WT and HPV/KO mice. In concordance with *in vivo* murine studies, demonstrating that dysregulated expression of the α2β1 integrin did not alter malignant conversion in SCC, α2β1 integrin expression in the K14-HPV16 model did not affect later aspects of tumor progression [48].

Although no difference in SCC progression was noted in *vivo,* when primary squamous carcinoma cells isolated from HPV/WT or HPV/KO mice were reintroduced orthotopically into either non-K14-HPV16 transgenic, wild-type or α2-null animals, the HPV/WT tumor cells, but not the HPV/KO tumor cells engrafted and grew rapidly. The HPV/WT tumor cells were significantly more migratory and invasive *in vitro*. Integrin loss on SCC cells resulted in reduced migration but even more striking deficiencies in invasion through collagen type I. [49], [50]. Our data suggest that α2β1 integrin-mediated interaction of squamous carcinoma cells with type I collagen, which is abundant in the dermis of mice and humans, may function to promote cancer cell migration and invasion, as seen in other models [49][[50]. HPV/KO tumor cells transfected with the wild-type mouse α2-integrin subunit failed to maintain integrin expression *in vivo*, thus preventing the analysis of integrin rescue in a SCC model of *in vivo* tumor formation and growth.

In patients with SCC, the development of lymph node metastasis is a predictor of poor outcome [51], [52]. Loss of the α2β1 integrin in the K14-HPV16 model resulted in decreased lymph node metastasis to regional lymph nodes by 31.3%. This correlates with an odds ratio of 1.7 for developing lymph node metastasis in the HPV/WT animals relative to HPV/KO mice. These data were quite surprising in light of our own recently published data that the α2β1 integrin acts as a tumor metastasis suppressor in breast and prostate cancer. In the mouse mammary tumor virus-Neu (MMTV-Neu) transgenic mouse model of breast cancer, lack of α2β1 integrin expression resulted in modestly decreased mammary tumor latency and markedly increased cancer metastasis [53].

The discordant contributions of the α2β1 integrin to metastasis in the HPV-stimulated model of SCC versus the neu-driven model of breast cancer raise interesting questions. First, the two models deal with distinctly different subtypes of carcinoma, SCC and adenocarcinoma, which arise from two disparate cells types. Perhaps the α2β1 integrin's role in regulating the multistep process of tumorigenesis is different depending on the cell of origin. Second, the metastatic route and mechanisms of dissemination are different between the two disease models. In the K14-HPV16 model, SCCs primarily metastasize via the lymphatics to regional lymph nodes. In the MMTV-neu model, cancer metastasizes primarily via the hematogenous route to the lungs. Third, the two tumor models are driven by different oncogenes that function distinctly. K14-HPV16 oncogenesis is triggered by expression of early region HPV16 oncogenes, E6 and E7, which inactivate two important tumor suppressor genes p53 and retinoblastoma, respectively. In contrast, the MMTV-Neu model of mammary cancer is driven by overexpression of the Neu tyrosine kinase that stimulates activation of the ras/map kinase cascade. Fourth, progression to carcinoma in the K14-HPV16 model requires the protumorigenic activity of inflammatory cells. Neither tumorigenesis nor metastasis in the MMTV-Neu model is dependent on recruitment of inflammatory cells. Fifth, the interaction of cancer cells with the specific collagen content of their microenvironment may be different. SCCs expressing the α2β1 integrin may have increased migration and invasion along collagen type I fibers in the skin, whereas receptor ligation with collagen type I may not be as important in breast cancer cell motility.

In summary, the α2β1 integrin plays a complex role in tumor progression through its contributions to both the malignant epithelial cell and within the tumor microenvironment. Our data are the first to suggest that integrin dependent regulation of tumor progression may be specific to the tissue type and to the mechanism of oncogenesis. In c-neu/HER2-positive breast cancer, the α2β1 integrin is a metastasis suppressor. In contrast, the α2β1 integrin promotes tumor metastasis in HPV-induced squamous cancer, likely by increasing the migratory and invasive ability of cells along collagen type I.

## Supporting Information

Figure S1 **The K14-HPV16 transgene, not the α2β1 integrin, mediates a robust inflammatory response.** *A–C*, Flow cytometric analysis of inflammatory cells was performed on the blood and preneoplastic ears of non-K14HPV16 transgenic wild-type (WT Ctrl) or α2-null (KO Ctrl) mice and HPV/WT and HPV/KO animals, either with (SCC^+^) or without tumors (SCC^−^). Similar analysis was also performed on the tumor tissue of HPV/WT and HPV/KO mice. The percentage of inflammatory cell subsets in HPV/WT and HPV/KO animals was compared to non-transgenic controls. Inflammation was highly dependent upon the presence of the K14-HPV16 transgene. Loss of the α2β1 integrin in HPV/KO ears increased the percentage of NK1.1^+^ cells relative to HPV/WT ears in non-tumor bearing animals (p = 0.014). Additionally, there was a significant increase in CD3ε^+^ T cells in HPV/KO tumor infiltrates, when compared to HPV/WT SCCs (p = 0.033). (Number of samples analyzed in blood and ear tissue: WT Ctrl n = 9; KO Ctrl n = 9; HPV/WT, SCC^+^ n = 12; HPV/WT, SCC^−^ n = 5; HPV/KO, SCC^+^ n = 14, HPV/KO, SCC^−^ n = 4. Number of samples analyzed in tumor tissue: HPV/WT, SCC^+^ n = 10 and HPV/KO, SCC^+^ n = 12.)
(TIF)

Figure S2 **Loss of the α2β1 integrin does not alter SCC growth, multiplicity, or grade.** *A*, Tumor volumes were measured weekly. The rate of tumor growth over time was calculated from tumor volume regression slopes and plotted as a function of time. No significant differences existed in the rates of SCC growth between HPV/WT (n = 22) and HPV/KO (n = 22) mice (p = 0.37). *B,* Total tumor burden for each HPV/WT (n = 97) and HPV/KO mouse (n = 73) was quantitated at the time of sacrifice. No significant differences were found for the multiplicity of tumor development (p = 0.45). *C,* Since multiple tumors may form on an animal, the highest grade scored was considered for analysis of differentiation loss. No significant differences were observed when considering the highest grade of SCC that developed in HPV/WT (n = 97) or HPV/KO (n = 73) mice (p = 0.57).
(TIF)

Figure S3 **In vitro proliferation of primary SCC cells was unaffected by loss of the α2β1 integrin.** Proliferation of the HPV/WT-1 and -2 and HPV/KO-1 and -2 SCC lines when adherent to collagen, fibronectin, or tissue culture plastic was determined *in vitro*. Proliferation *in vitro* of HPV/WT and HPV/KO lines was similar irrespective of the matrix (p = 0.35, p = 0.33, p = 0.42, respectively).
(TIF)

Table S1 **Detailed Analysis of Inflammatory Cell Populations in Blood, Preneoplastic Ears, and Tumors.** WT Ctrl and KO Ctrl animals were used to verify and establish baseline inflammatory populations independent of the K14-HPV16 transgene. Chi^2^ probability with ties analysis was performed on all 6 groups for each specific tissue; those found to be significant or close to p<0.05 were analyzed further through inter-comparison of the 6 groups by Mann-Whitney tests. The groups in which significance was found are denoted as Genotype 1 vs. Genotype 2. Differences in inflammatory cells were found between non-K14-HPV16 transgenic, control animals and those expressing the K14-HPV16 transgene. Integrin-dependent differences were identified in the NK1.1-positive and CD3ε-positive cell populations. Non-neoplastic ear tissue in HPV/KO, SCC^−^ mice had increased NK1.1-positive cells than HPV/WT, SCC^−^ ears (p = 0.014). HPV/KO SCCs contained more CD3ε-positive cells than HPV/WT tumors (p = 0.033). T regulatory cells were defined as CD4, CD25, and Foxp3 triple-positive cells as a percentage of CD4-positive cells. (Blood and ear samples analyzed: WT Ctrl n = 9; KO Ctrl n = 9; HPV/WT, SCC^+^ n = 12, HPV/WT, SCC^−^ n = 5; HPV/KO, SCC^+^ n = 14, HPV/KO, SCC^−^ n = 4. Tumor tissue analyzed: HPV/WT, SCC^+^ n = 10 and HPV/KO, SCC^+^ n = 12). * represents p<0.05 ** represents p<0.001 *** represents p<0.0001.
(DOCX)
