# Supplementary figures and images for: Loss of the α2β1 Integrin Alters Human Papilloma Virus-Induced Squamous Carcinoma Progression In Vivo and In Vitro
Source: PLoS One. 2011 Oct 27;6(10):e26858. doi: 10.1371/journal.pone.0026858 (PMC3203166; doi:10.1371/journal.pone.0026858)

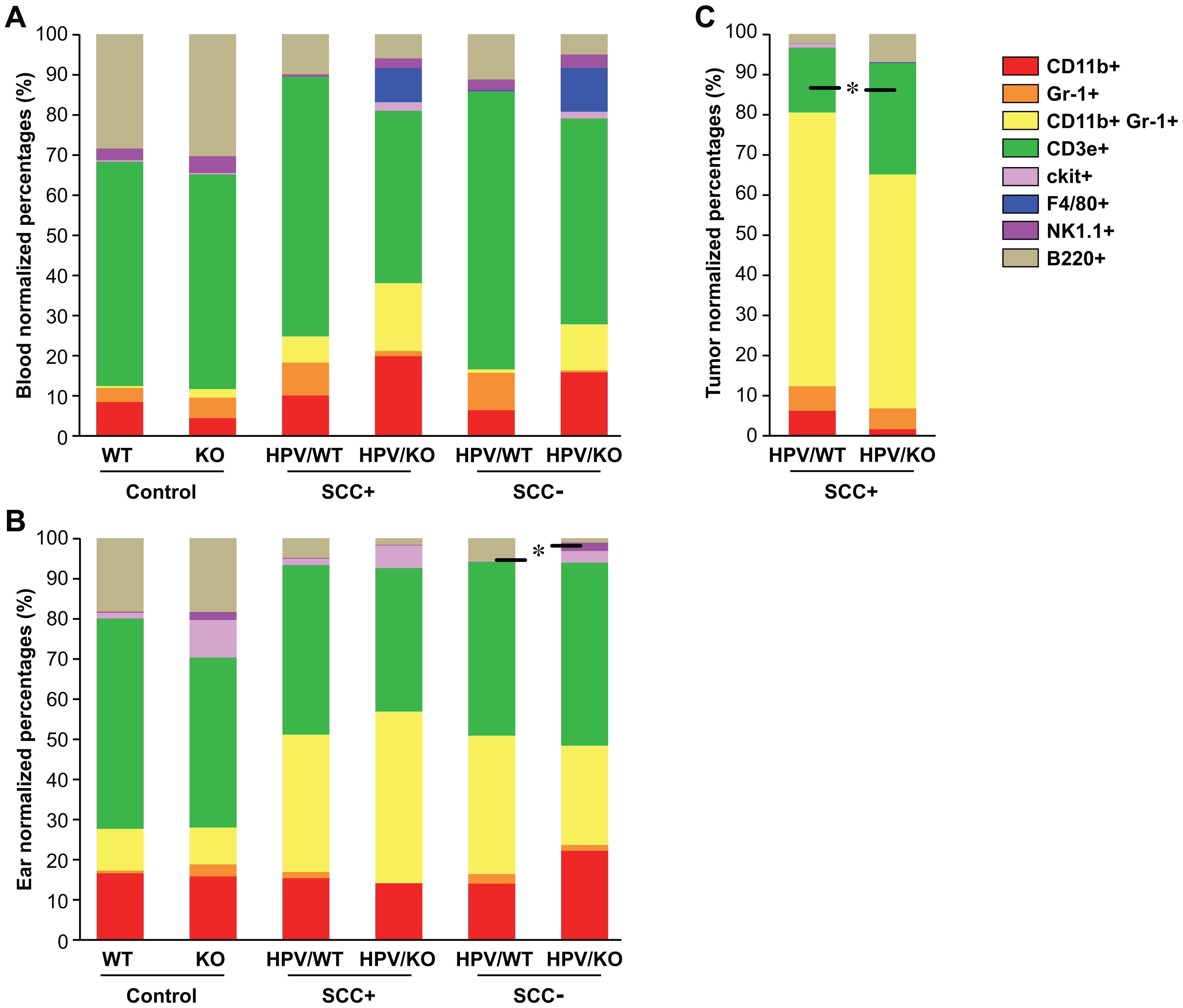

Supplement: Figure S1 — The K14-HPV16 transgene, not the α2β1 integrin, mediates a robust inflammatory response. A–C, Flow cytometric analysis of inflammatory cells was performed on the blood and preneoplastic ears of non-K14HPV16 transgenic wild-type (WT Ctrl) or α2-null (KO Ctrl) mice and HPV/WT and HPV/KO animals, either with (SCC+) or without tumors (SCC−). Similar analysis was also performed on the tumor tissue of HPV/WT and HPV/KO mice. The percentage of inflammatory cell subsets in HPV/WT and HPV/KO animals was compared to non-transgenic controls. Inflammation was highly dependent upon the presence of the K14-HPV16 transgene. Loss of the α2β1 integrin in HPV/KO ears increased the percentage of NK1.1+ cells relative to HPV/WT ears in non-tumor bearing animals (p = 0.014). Additionally, there was a significant increase in CD3ε+ T cells in HPV/KO tumor infiltrates, when compared to HPV/WT SCCs (p = 0.033). (Number of samples analyzed in blood and ear tissue: WT Ctrl n = 9; KO Ctrl n = 9; HPV/WT, SCC+ n = 12; HPV/WT, SCC− n = 5; HPV/KO, SCC+ n = 14, HPV/KO, SCC− n = 4. Number of samples analyzed in tumor tissue: HPV/WT, SCC+ n = 10 and HPV/KO, SCC+ n = 12.) (TIF) [file pone.0026858.s001.tif]

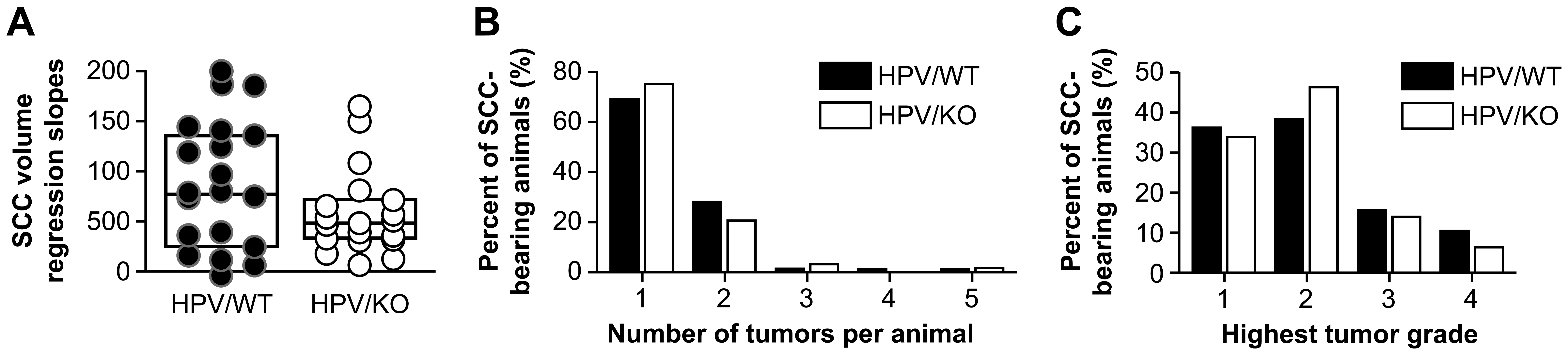

Supplement: Figure S2 — Loss of the α2β1 integrin does not alter SCC growth, multiplicity, or grade. A, Tumor volumes were measured weekly. The rate of tumor growth over time was calculated from tumor volume regression slopes and plotted as a function of time. No significant differences existed in the rates of SCC growth between HPV/WT (n = 22) and HPV/KO (n = 22) mice (p = 0.37). B, Total tumor burden for each HPV/WT (n = 97) and HPV/KO mouse (n = 73) was quantitated at the time of sacrifice. No significant differences were found for the multiplicity of tumor development (p = 0.45). C, Since multiple tumors may form on an animal, the highest grade scored was considered for analysis of differentiation loss. No significant differences were observed when considering the highest grade of SCC that developed in HPV/WT (n = 97) or HPV/KO (n = 73) mice (p = 0.57). (TIF) [file pone.0026858.s002.tif]

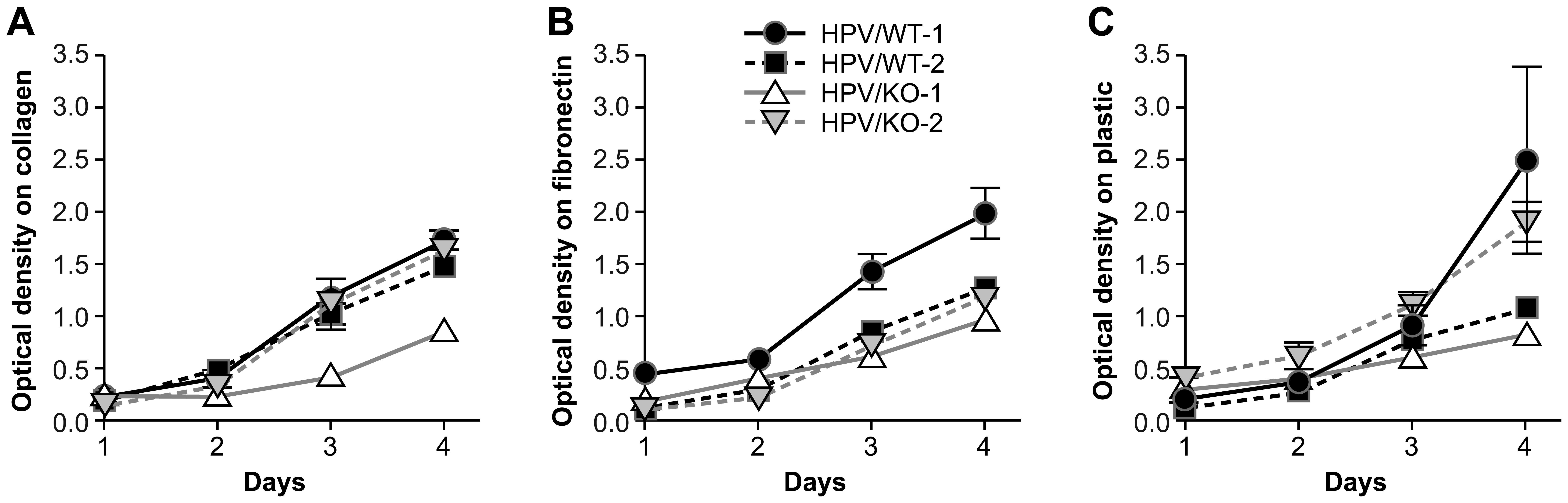

Supplement: Figure S3 — In vitro proliferation of primary SCC cells was unaffected by loss of the α2β1 integrin. Proliferation of the HPV/WT-1 and -2 and HPV/KO-1 and -2 SCC lines when adherent to collagen, fibronectin, or tissue culture plastic was determined in vitro. Proliferation in vitro of HPV/WT and HPV/KO lines was similar irrespective of the matrix (p = 0.35, p = 0.33, p = 0.42, respectively). (TIF) [file pone.0026858.s003.tif]
