# Supplementary material for: Loss of the α2β1 Integrin Alters Human Papilloma Virus-Induced Squamous Carcinoma Progression In Vivo and In Vitro
Source: PLoS One. 2011 Oct 27;6(10):e26858. doi: 10.1371/journal.pone.0026858 (PMC3203166; doi:10.1371/journal.pone.0026858)
Supplement: Table S1 — Detailed Analysis of Inflammatory Cell Populations in Blood, Preneoplastic Ears, and Tumors. WT Ctrl and KO Ctrl animals were used to verify and establish baseline inflammatory populations independent of the K14-HPV16 transgene. Chi2 probability with ties analysis was performed on all 6 groups for each specific tissue; those found to be significant or close to p<0.05 were analyzed further through inter-comparison of the 6 groups by Mann-Whitney tests. The groups in which significance was found are denoted as Genotype 1 vs. Genotype 2. Differences in inflammatory cells were found between non-K14-HPV16 transgenic, control animals and those expressing the K14-HPV16 transgene. Integrin-dependent differences were identified in the NK1.1-positive and CD3ε-positive cell populations. Non-neoplastic ear tissue in HPV/KO, SCC− mice had increased NK1.1-positive cells than HPV/WT, SCC− ears (p = 0.014). HPV/KO SCCs contained more CD3ε-positive cells than HPV/WT tumors (p = 0.033). T regulatory cells were defined as CD4, CD25, and Foxp3 triple-positive cells as a percentage of CD4-positive cells. (Blood and ear samples analyzed: WT Ctrl n = 9; KO Ctrl n = 9; HPV/WT, SCC+ n = 12, HPV/WT, SCC− n = 5; HPV/KO, SCC+ n = 14, HPV/KO, SCC− n = 4. Tumor tissue analyzed: HPV/WT, SCC+ n = 10 and HPV/KO, SCC+ n = 12). * represents p<0.05 ** represents p<0.001 *** represents p<0.0001. (DOCX) [file pone.0026858.s004.docx]

**Table S1. Detailed Analysis of Inflammatory Cell Populations in Blood, Preneoplastic Ears, and Tumors.**

| **Tissue** | **Marker** | **Chi^2^ Probability with Ties** | **Genotype 1** | **vs.** | **Genotype 2** | **Mann-Whitney Test** |
| --- | --- | --- | --- | --- | --- | --- |
| Blood | CD11b | 0.56 |  |  |  |  |
| Blood | Gr-1 | 0.010* | KO Ctrl | > | HPV/KO, SCC- | 0.0055** |
| Blood | Gr-1/CD11b | 0.17 |  |  |  |  |
| Blood | CD3ε | 0.58 |  |  |  |  |
| Blood | ckit | 0.79 |  |  |  |  |
| Blood | F4/80 | 0.057 | WT Ctrl | < | HPV/WT, SCC- | 0.02* |
| Blood |  |  | KO Ctrl | < | HPV/KO, SCC- | 0.031* |
| Blood | NK1.1 | 0.0030* | KO Ctrl | > | HPV/KO, SCC+ | 0.0019** |
| Blood | B220 | 0.0001* | WT Ctrl | > | HPV/WT, SCC+ | 0.0056** |
| Blood |  |  | WT Ctrl | > | HPV/WT, SCC- | 0.014* |
| Blood |  |  | KO Ctrl | > | HPV/KO, SCC+ | 0.0003*** |
| Blood |  |  | KO Ctrl | > | HPV/KO, SCC- | 0.0055** |
| Blood | CD4 | 0.19 |  |  |  |  |
| Blood | % Tregs of CD4+ | 0.023* | WT Ctrl | < | HPV/WT, SCC+ | 0.055 |
| Blood |  |  | KO Ctrl | < | HPV/KO, SCC+ | 0.025* |
| Ear | CD11b | .17 |  |  |  |  |
| Ear | Gr-1 | .77 |  |  |  |  |
| Ear | Gr-1/CD11b | .0011** | WT Ctrl | < | HPV/WT, SCC+ | 0.011* |
| Ear |  |  | WT Ctrl | < | HPV/WT, SCC- | 0.014* |
| Ear |  |  | KO Ctrl | < | HPV/KO, SCC+ | 0.0033** |
| Ear |  |  | KO Ctrl | < | HPV/KO, SCC- | 0.031* |
| Ear | CD3ε | .17 |  |  |  |  |
| Ear | ckit | .83 |  |  |  |  |
| Ear | F4/80 | .077 |  |  |  |  |
| Ear | NK1.1 | .03* | KO Ctrl | > | HPV/KO, SCC+ | 0.037* |
| Ear |  |  | HPV/WT, SCC- | < | HPV/KO, SCC- | 0.014* |
| Ear | B220 | .012* | WT Ctrl | > | HPV/WT, SCC+ | 0.028* |
| Ear |  |  | KO Ctrl | > | HPV/KO, SCC- | 0.0087** |
| Ear | CD4 | .15 |  |  |  |  |
| Ear | % Tregs of CD4+ | .0058** | KO Ctrl | < | HPV/KO, SCC+ | 0.049* |
| Ear |  |  | KO Ctrl | > | HPV/KO, SCC- | 0.021* |
| Tumor | CD11b |  | HPV/WT, SCC+ | ≈ | HPV/KO, SCC+ | .80 |
| Tumor | Gr-1 |  | HPV/WT, SCC+ | ≈ | HPV/KO, SCC+ | .87 |
| Tumor | Gr-1/CD11b |  | HPV/WT, SCC+ | ≈ | HPV/KO, SCC+ | 1.0 |
| Tumor | CD3ε |  | HPV/WT, SCC+ | < | HPV/KO, SCC+ | .033* |
| Tumor | ckit |  | HPV/WT, SCC+ | ≈ | HPV/KO, SCC+ | .66 |
| Tumor | F4/80 |  | HPV/WT, SCC+ | ≈ | HPV/KO, SCC+ | .66 |
| Tumor | NK1.1 |  | HPV/WT, SCC+ | ≈ | HPV/KO, SCC+ | .84 |
| Tumor | B220 |  | HPV/WT, SCC+ | ≈ | HPV/KO, SCC+ | .27 |
| Tumor | CD4 |  | HPV/WT, SCC+ | ≈ | HPV/KO, SCC+ | .32 |
| Tumor | % Tregs of CD4+ |  | HPV/WT, SCC+ | ≈ | HPV/KO, SCC+ | .11 |
